# Aqueous chemimemristor based on proton-permeable graphene membranes

**DOI:** 10.1073/pnas.2314347121

**Published:** 2024-02-01

**Authors:** Yongkang Wang, Takakazu Seki, Paschalis Gkoupidenis, Yunfei Chen, Yuki Nagata, Mischa Bonn

**Affiliations:** ^a^Jiangsu Key Laboratory for Design and Manufacture of Micro-Nano Biomedical Instruments, School of Mechanical Engineering, Southeast University, Nanjing 211189, China; ^b^Molecular Spectroscopy Department, Max Planck Institute for Polymer Research, Mainz 55128, Germany

**Keywords:** aqueous memristor, iontronics, proton permeation, graphene, HD-SFG spectroscopy

## Abstract

Memristive devices, electrical elements whose resistance depends on the history of applied electrical signals, are leading candidates for future data storage and neuromorphic computing. Memristive devices typically rely on solid-state technology, while aqueous memristive devices are crucial for biology-related applications such as next-generation brain-machine interfaces. Here, we report a simple graphene-based aqueous memristive device with long-term and tunable memory regulated by reversible voltage-induced interfacial acid-base equilibria enabled by selective proton permeation through the graphene. Surface-specific vibrational spectroscopy verifies that the memory of the graphene resistivity arises from the hysteretic proton permeation through the graphene, apparent from the reorganization of interfacial water at the graphene/water interface. The proton permeation alters the surface charge density on the CaF_2_ substrate of the graphene, affecting graphene’s electron mobility, and giving rise to synapse-like resistivity dynamics. The results pave the way for developing experimentally straightforward and conceptually simple aqueous electrolyte-based neuromorphic iontronics using two-dimensional (2D) materials.

## Significance

We implemented memristive behavior in aqueous electrolytes using monolayer graphene supported on a CaF_2_ substrate as a model system and probed in situ the ion dynamics of the neuromorphic devices with surface-specific vibrational spectroscopy. Our work demonstrates a unique and simple concept for developing aqueous electrolyte-based neuromorphic iontronics using two-dimensional (2D) materials through manipulating interfacial acid-base equilibrium.

Conventional computers based on von Neumann’s architecture operate mostly sequentially. Neuromorphic computing uses hardware-based implementations to mimic the behavior of synapses and neurons in the brain, allowing for efficient brain-inspired computing in a massively parallel fashion. It is more efficient for sophisticated computational tasks such as artificial cognition and intelligence (1–6). Synaptic plasticity and the Hebbian learning principle are thought to form the basis of learning and memory and are primarily responsible for information processing in the brain (1, 7–9). As such, developing devices and circuits that can display synaptic functionality (1, 10) is crucial for advancing neuromorphic computing.

Memristive devices, whose resistance/conductance states depend on the history of applied electrical signals states, can display such synaptic functionality and have been important candidates for neuromorphic computing (4, 5, 11–15). Extensive efforts have been devoted to their development and optimization primarily based on solid-state devices (like the metal-insulator-metal or MIM architecture) (16, 17). Artificial systems capable of implementing the synaptic functionality in aqueous electrolytes, using ions in water as charge carriers, have received much less attention (9, 18–24). Such aqueous memristive devices are analogs of biological synapses. Developing water-based bioinspired memristive devices is significant for neuromorphic computing and developing next-generation brain-machine interfaces (2, 13, 25–28). Several aqueous memristive devices have previously been developed. For example, organic electrochemical devices using aqueous electrolytes provide memory with low-voltage operation (<150 mV) and sensitive response to typical physiological and pathological ionic concentration ranges (5 to 150 mM) (20–22). Recently, nanofluidic devices have also been reported in which solvated ion transport exhibits memristive behavior (9, 18). The challenge associated with these approaches is the complexity of the device fabrication (21, 29, 30). Realizing memristive behavior in a simple system is highly desirable. Following this motivation, we here proposed a relatively simple aqueous proton-based memristive device based on a calcium fluoride (CaF_2_)-supported monolayer graphene in contact with bulk water. The device design is enabled by molecular-level insights into the memristive ion/water dynamics and the corresponding synaptic phenomena in this aqueous memristive device. The memristive behavior arises from the fast proton transfer across the graphene and the relatively slow diffusion process of protons. Despite the device’s simplicity, this aqueous device exhibits long-term and tunable memory (from 60 to 6,000 s) and promising potential for large-scale integration and multiplication.

Among different potential ions for aqueous memristive devices, protons are particularly interesting, having been recognized as essential charge carriers regulating synaptic plasticity (31–33). Moreover, protons are intrinsic to water, being an auto-dissociation product whose concentration determines the pH. Protons exhibit anomalously high mobility in water enabled by the Grotthus mechanism of proton transport (34). Protons can tunnel through graphene, giving rise to selective proton permeation (35–37). Graphene is readily incorporated into electronic devices and is a superior chemical-, and particularly pH-sensing resistor (38–40) possessing unparalleled breaking strength, ultra-high chemical stability, ambipolar conductance (41), and pH-sensitive conductance (42), making it a promising candidate for developing aqueous proton-based memristive devices (43).

Here, we show the simple CaF_2_-supported monolayer graphene in contact with bulk water exhibits long-term and tunable memory. Uniquely, the graphene acts as the agent, both changing the pH by reversible local electrochemistry, and reporting the local pH through its pH-modified conductivity, hence the term *chemimemristor*. In addition to the electrical characterization of the device, we probe the interfacial water arrangement at the graphene/water interface employing heterodyne-detected sum-frequency generation (HD-SFG) spectroscopy. This technique is ideal for providing insightful information not only on the structure and orientation of interfacial water molecules (*up*-/*down*-orientation) (44–49), but also on the ion arrangement and dynamics at the interface (50–53). Combining electrical measurements with HD-SFG lets us fully map the ion dynamics at the graphene/water interface and determine the mechanism underlying the aqueous electrolyte-based graphene memristive device. Our work demonstrates a unique and simple concept for developing aqueous electrolyte-based neuromorphic iontronics using two-dimensional (2D) materials through manipulating interfacial acid-base equilibrium. The method and findings presented here also pave the way for resolving molecular-level details of the ion-mediated synaptic behavior in aqueous electrolyte-based neuromorphic iontronics.

## Results and Discussion

### Memristive Effect of the Aqueous Proton-based Memristive Device.

**Fig. 1.**
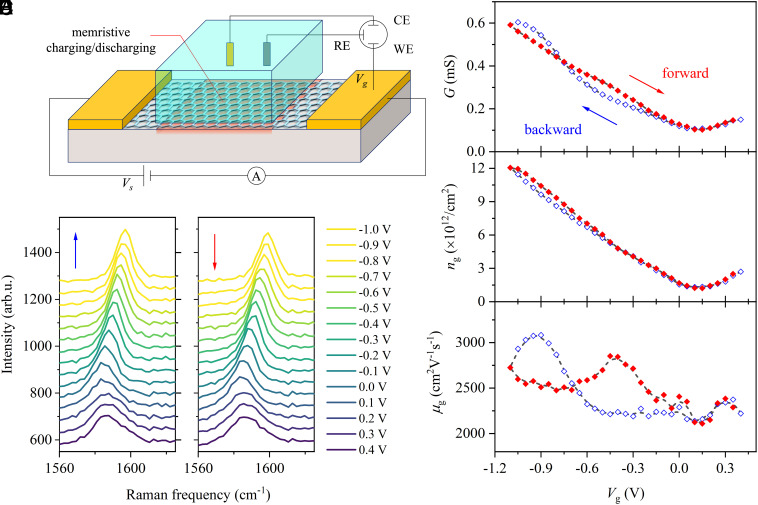
Memristive effect of the aqueous proton-based memristive device made of CaF_2_-supported monolayer graphene. (*A*) Schematic diagram of the memristive device. The red region of the CaF_2_ substrate in the schematic diagram indicates the charging/discharging region exposed to the aqueous protons. Measurements were conducted at a flow rate of 50 µm/s, a scan rate of 5 mV/s, and a step of 50 mV. (*B*) G as a function of $V_{g}$. The open blue diamonds and the solid red diamonds indicate the backward and forward scan, respectively. (*C*) Raman spectra at various $V_{g}$. The G-band frequency reaches a minimum at the charge-neutral point around 0.1 V vs. Pd/H_2_ and blueshifts as a function of $V_{g}$. The spectra are offset for clarity. The *Left* and *Right* panels correspond to the backward and forward scans. (*D*) $n_{g}$ obtained from the Raman G-band frequency shift as a function of $V_{g}$. (*E*) Inferred $\mu_{g}$ as a function of $V_{g}$.

Our device is an aqueous electrolyte-gated monolayer graphene field-effect transistor (GFET), similar to previous solid-state graphene-based memristive devices for neuromorphic computing (54–56). Fig. 1*A* shows a schematic diagram of the GFET. To fabricate the GFET, large-area chemical vapor deposition (CVD)-grown graphene was transferred onto a calcium fluoride (CaF_2_) substrate on which two gold strips were pre-deposited. The two gold strips serve as the source and drain electrodes for measuring the graphene’s conductance (G). We used 10 mM NaClO_4_ aqueous solution (pH ~ 7) as the gate electrolyte. The gate potentials ($V_{g}$) on the graphene were applied against a Pd/H_2_ reference electrode, which functions as a top-gate electrode. Furthermore, to ensure accurate potential control on the graphene electrode, a gold wire was employed to construct a typical three-electrode system. Monolayer graphene, Pd/H_2_, and the gold wire serve as the working electrode (WE), reference electrode (RE, top-gate electrode), and counter electrode (CE), respectively. These three electrodes were connected to an electrochemical workstation (Metrohm Autolab PGSTAT302). Flowing the electrolyte solution weakens memory effects (49, 57) but avoids the change of pH of the NaClO_4_ aqueous solution during our electrochemical measurements. The electrolyte solution was flown through the cell at a constant flow rate (50 μm/s, defined as the measured water volume flowing per unit of time divided by the cross-section of the flow channel). Further details regarding the device fabrication, characterization, and measurements can be found in *SI Appendix*, section S1 and *Methods*.

We first characterize the electrical responses of the CaF_2_-supported GFET by measuring G upon applying $V_{g}$. G was determined from source-drain current ($I_{SD}$) at a constant bias ($V_{SD}=50\;\text{mV}\;$) using Ohm’s law $G=I_{SD}/V_{SD}$. The data as a function of $V_{g}$ were displayed in Fig. 1*B*. G reaches a minimum at the charge-neutral point around 0.1 V and increases as a function of $V_{g}$. Markedly, the $G-V_{g}$ curve displays hysteretic changes and a self-crossing point at around −0.8 V, which indicates that the CaF_2_-supported graphene/water system displays memristive behavior (58, 59). Notably, this memristive effect was found to take place at frequencies between 0.17 and 17 mHz (*SI Appendix*, section S2), corresponding to memory timescales from tens (~60 s) to thousands of seconds (~6,000 s), well below frequencies where capacitive effects (the time constant of the electric double layer is ~microseconds) can introduce hysteresis. The memristive effect shows no dependence on the size of the graphene, suggesting its potential for large-scale integration and multiplication (*SI Appendix*, section S3). We also stress that the memristive effect is highly reproducible for different graphene devices and is weakly affected by presence of defects in graphene (*SI Appendix*, section S1).

The graphene conductance is related to its charge carrier density ($n_{g}$) and mobility ($\mu_{g}$) via

[1] $$G=n_{g}e\mu_{g}+G_{min},$$

where e is the elementary charge, $G_{min}$ is the minimum conductivity which changes negligibly with $V_{g}$ (*SI Appendix*, section S4). A question is whether this memristive behavior arises from the variation of $n_{g}$ or the variation of $\mu_{g}$ with applied $V_{g}$ (60). To examine this, we independently determine $n_{g}$ from Raman G-band frequency shift of the graphene electrode (61, 62) (*SI Appendix*, section S5). The Raman G-band data are shown in Fig. 1*C*, while the inferred $n_{g}$ is shown in Fig. 1*D*. $n_{g}$ varies nearly linearly with $V_{g}$ and displays negligible hysteresis. Other factors such as mechanical strain (63) and ion adsorption (64, 65) on the graphene could also induce the G-band frequency shift, but their effects are minor and were ignored in this study (*SI Appendix*, section S5).

Knowing $n_{g}$, we extract $\mu_{g}$ by using Eq. **1**. $\mu_{g}$ vs. $V_{g}$ are shown in Fig. 1*E*. Unlike $n_{g}$, the $\mu_{g}-V_{g}$ curve displays a remarkable self-crossing loop, with the lowest and highest values significantly differing by a factor of 1.3 ~ 2.0. $\mu_{g}$ shows negligible changes with a value of around 2,200 ${cm}^{2}V^{-1}s^{-1}$ when backward scanning the potentials from 0.3 to −0.5 V, and then rapidly increases to a maximum of 3,100 $m^{2}V^{-1}s^{-1}$ between −0.5 V and −0.9 V. Below −0.9 V, the $\mu_{g}$ drops down to 2,700 $m^{2}V^{-1}s^{-1}$ at −1.1 V. Forward scanning the potential, the nonlinear change of $\mu_{g}$ reverses, and the $\mu_{g}$ is reset to its initial value, but in a significantly hysteretic manner. Our result reveals that the observed memristive effect in graphene conductance arises from the hysteretic change of $\mu_{g}$.

### Hysteresis of $\mu_{g}$ Is Due to Charged-Impurity Scattering.

**Fig. 2.**
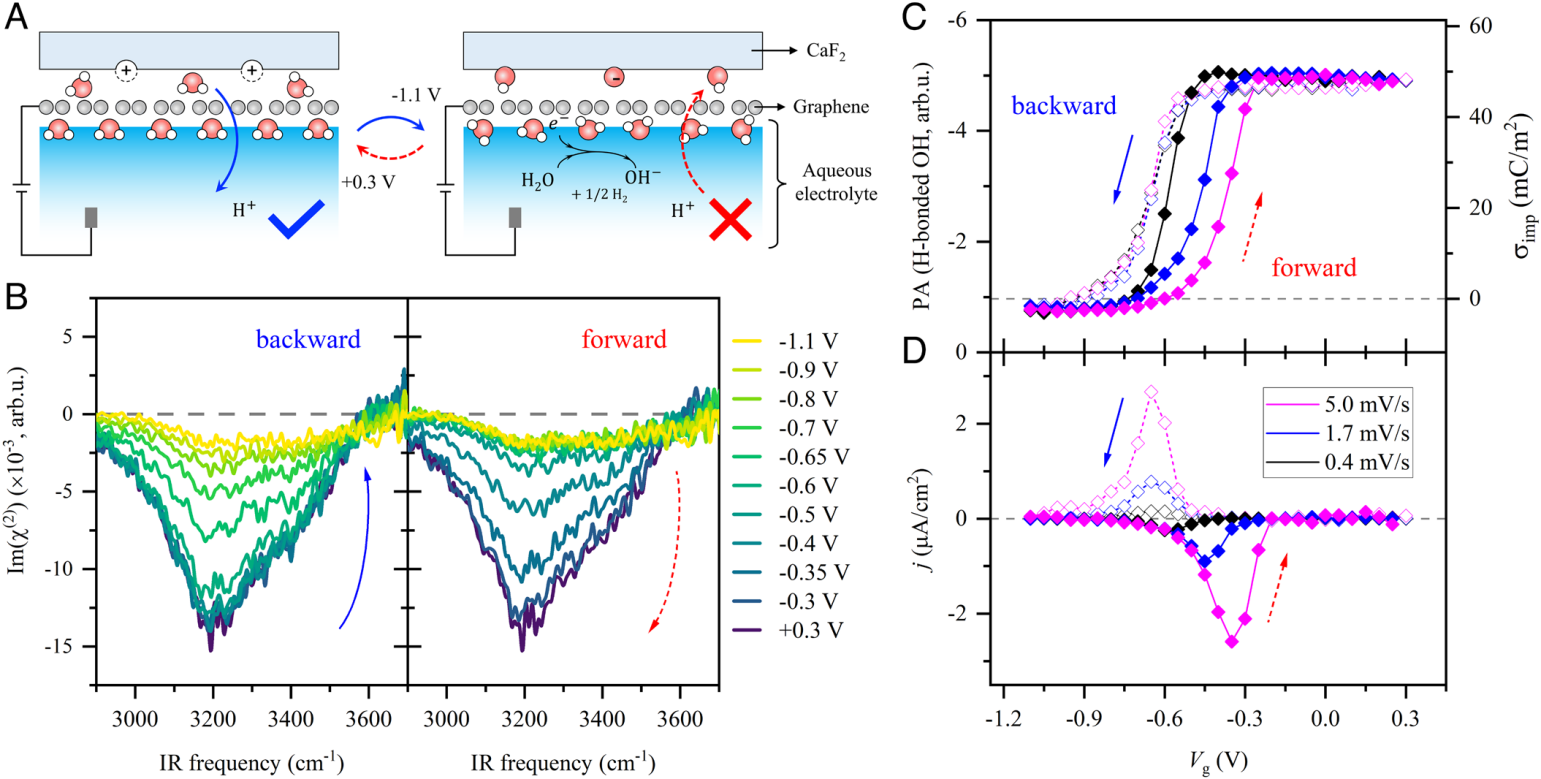
Memristive charging/discharging of CaF_2_, revealed by HD-SFG spectroscopy. We used 10 mM NaClO_4_ as the gating electrolyte. The flow rate is 50 µm/s. (*A*) Schematic diagram of the memristive charging/discharging of the CaF_2_ substrate enabled by aqueous proton permeation through the graphene electrode. (*B*) The O–H stretching $Im\left(\chi^{\left(2\right)}\right)$ spectra at the CaF_2_-supported graphene/water interface at various $V_{g}$. The potential scan rate is 5 mV/s. The *Left* and *Right* panels correspond to the backward and forward potential scans, respectively. The dashed lines indicate the zero line. (*C*) Peak amplitudes (PA) in the $Im\left(\chi^{\left(2\right)}\right)$ spectra as a function of $V_{g}$ at various potential scan rates. The open and solid diamonds indicate the backward and forward scans, respectively. The gray dashed line indicates the zero charge. (*D*) Corresponding current density j as a function of $V_{g}$. The gray dashed line indicates the zero line.

A question arising here is how the hysteretic change of $\mu_{g}$ occurs. A probable explanation is that the number of charged impurities ($\sigma_{imp}$, which refers to the surface charges on CaF_2_) changes, which then affects $\mu_{g}$ through long-range Coulomb scattering (for $\sigma_{imp}<∼1.6\;\text{mC}/m^{2}$, $\mu_{g}\propto 1/\sigma_{imp}$) (60, 66). This is consistent with previously reported behavior in which the pseudocapacitive charging/discharging of the CaF_2_ substrate occurs when the graphene is electrified due to water dissociation-induced local pH change (49, 57) (Fig. 2*A* and *SI Appendix*, section S6). If $\sigma_{imp}$ modifies the graphene conductance, it should also show the hysteresis profile with a memory effect. To test this hypothesis, we measured $\sigma_{imp}$ through the degree of water alignment at the CaF_2_-supported graphene/water interface reflected in the HD-SFG signal $\left(Im\left(\chi^{\left(2\right)}\right)\right)$ of the interfacial water in the O–H stretch mode region (2,900 to 3,700 cm^−1^) (50, 51, 67). Water alignment at the CaF_2_-supported graphene electrode/water interface is dominated by the variation of surface charges on CaF_2_ (σ_imp_) (49, 57, 68). We can thus infer $\sigma_{imp}$ from the HD-SFG signal of the interfacial water.

The $Im\left(\chi^{\left(2\right)}\right)$ spectra at various $V_{g}$ with a scan rate of 5 mV/s are shown in Fig. 2*B*. The negative hydrogen-bonded (H-bonded) O–H peak (2,950 to 3,550 cm^−1^) indicates that the O–H group of the interfacial water points down toward the bulk water. The negative signal is weakened upon decreasing $V_{g}$ because the surface charge of the CaF_2_ substrate decreases.

We quantify the variation of the $Im\left(\chi^{\left(2\right)}\right)$ signal by integrating the peak area (PA) of the H-bonded O–H peak as a measure of the surface charge on the CaF_2_ substrate ($\sigma_{imp}\propto PA$; for details, see *SI Appendix*, section S7). The data are depicted in Fig. 2*C*. $\sigma_{imp}$ exhibits a memristive change upon changing $V_{g}$. It rapidly and nonlinearly decreases from positive to negative during backward scanning the potentials from 0.3 to −1.1 V and reverses its changes when forward scanning the potential but with a significant hysteresis. The nonlinear and hysteretic changes of $\sigma_{imp}$ are consistent with the changes of $\mu_{g}$ ($\mu_{g}\propto 1/\sigma_{imp}\propto 1/PA$, see *SI Appendix*, section S8). Backward scanning the potentials from 0.3 to −0.5 V, both $\sigma_{imp}$ and $\mu_{g}$ are constant. Lowering $V_{g}$ from −0.5 to −0.9 V, $\sigma_{imp}$ rapidly and nonlinearly decreases, explaining the nonlinear increase of $\mu_{g}$. With a further decrease of $V_{g}$, $\sigma_{imp}$ becomes negative and increases, explaining the subsequent decrease of $\mu_{g}$. This consistency also holds for the forward scanning direction (*SI Appendix*, section S8). The memristive charging/discharging processes are highly reproducible and general, i.e., independent of the electrolyte and details of the 2D material: We observe memristive behavior in devices made of CaF_2_-supported double-layer graphene, CaF_2_-supported graphene/hexagonal boron nitride (hBN) heterostructures, and fused silica (SiO_2_)-supported monolayer graphene. Detailed results can be found in *SI Appendix*, sections S9–S11.

### Memory Originates from H^+^ Transport Across Graphene.

To obtain molecular-level insights into the memristive charging/discharging of CaF_2_, we performed SFG measurements at different scan rates ($v_{s}$). The PA and $\sigma_{imp}$ at different $v_{s}$ are shown in Fig. 2*C*. $v_{s}$ hardly affects the nonlinear decrease of $\sigma_{imp}$ in the backward scan but greatly affects its variation in the forward scan. The memory effects are weakened by decreasing $v_{s}$ and very small at $v_{s}=0.4$ mV/s. These results imply that the observed memory effects in our aqueous proton-based memristive device relate to the memristive charging process of the CaF_2_ substrate.

The charging/discharging of the CaF_2_ substrate can be described via (49)

[I] $$\equiv {Ca}^{+}\cdots H_{2}O⇌\equiv CaOH+H^{+},$$

[II] $$H^{+}\left({CaF}_{2}/graphene\right)⇌H^{+}\left(graphene/water\right),$$

where ≡ indicates a surface-bound state. The CaF_2_ substrate is positively charged at neutral pH (~7), with its isoelectric point in the pH range of 9 to 10 (69, 70). The pseudocapacitive response of the electrochemical system triggers a local pH increase for increasingly negative potential, neutralizing the surface charge following reaction (I). Such a reaction is enabled by the tiny amount of trapped water between the graphene and the CaF_2_ substrate during sample preparation (49). We note that the distribution of water trapped between graphene and CaF_2_ substrate is uniform and does not affects charging/discharging of the CaF_2_ substrate and thus the performance of our aqueous proton-based memristive device (*SI Appendix*, sections S1 and S6).

Inversely, in the forward scan, the potential increases with time, and the chemical equilibrium of (I) tends to the left. This reaction requires proton transfer from the bulk water to the CaF_2_/graphene interface. The hysteretic charging of the CaF_2_ during the forward scan suggests a slow kinetic of (II) to the left. The slow kinetic is not due to the energy barrier for protons to transfer through the graphene (*SI Appendix*, section S12). Rather, it is because of diffusion-limited proton transport from bulk water to the interface (49, 71, 72). Accordingly, a faster bulk supply rate of protons by increasing the flow rate weakens the memory effect. Increasing the electrolyte concentration strengthens the memory effect (73), also in line with this hypothesis (*SI Appendix*, section S12). As such, the kinetics of charging the CaF_2_ is governed by step (II).

In contrast, in the backward scan, the surface charge on the CaF_2_ surface changes immediately through chemical equilibrium (I) driven by the applied potentials. Thus, the memory effect disappears in the backward scan. Consequently, the characteristic memory effect in our graphene-based aqueous proton memristive device is associated with the memristive proton permeation through the graphene electrode (Fig. 2*D*). Such memristive proton permeation is also observed in CV measurements (*SI Appendix*, section S6). Furthermore, these results also suggest that the memory of our memristive device can be readily tuned by changing the potential scan rate (*SI Appendix*, section S2).

### Synaptic Plasticity and State-Retention.

**Fig. 3.**
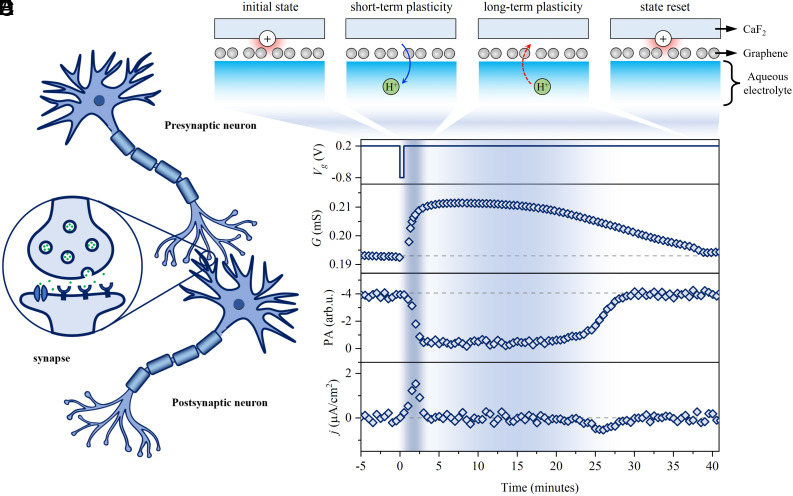
Synaptic plasticity and state-retention. (*A*) Diagram of the biological neural network. The inset shows the single neuron synapse. Protons serve as the neurotransmitter (green dots), allowing for communication between the presynaptic neuron and the post-synaptic neuron. (*B*) Sketch diagram showing the correspondence of proton permeation and state-retention of the graphene memristive device. (*C*) Change of G as a function of time after a −0.8 V spike (30 s). The operation gate potential (baseline for the spike) is 0.2 V. Waveform of the spike is shown in the *Top* panel. The flow rate is 10 µm/s. (*D* and *E*) Peak amplitudes (PA) in the $Im\left(\chi^{\left(2\right)}\right)$ spectra and corresponding current density j as a function of time. The dark and light blue shadow areas in *C*, *D*, and *E* indicate the short- and long-term state-retention, respectively. The dashed lines in *C*, *D*, and *E* indicate the corresponding initial state.

The above HD-SFG measurements reveal that the long-term memory and biological synapse-like dynamics of proton permeation through the graphene underlies the conductance switching mechanism in our aqueous proton-based memristive device. Protons regulate the synaptic response of the memristive device (Fig. 3 *A* and *B*). To verify this, we characterized the state-retention of our graphene memristive device, a fundamental synaptic plasticity functionality that is the basis for neuromorphic computing (1, 7–9). The data are shown in Fig. 3*C*. Following a −0.8 V spike, G rapidly increases and quickly relaxes in a short period (∼5 min) to a long-term value (~20 min), demonstrating that our memristive device displays both short- and long-term memory, corresponding to short- and long-term synaptic plasticity (9, 74, 75). The HD-SFG measurements reveal that the short-term synaptic plasticity can be correlated to the discharging of CaF_2_ (Fig. 3*D*) accompanied by proton permeation to the bulk water (the sharp positive j peak in Fig. 3*E*). Discharging of CaF_2_ raises $\mu_{g}$ and thus G (for details, see *SI Appendix*, section S13). While the long-term synaptic plasticity results from the hysteric proton permeation from bulk water to the CaF_2_/graphene interface, manifesting as a near-zero j. After a long-term relaxation (~20 min), G is reset to its initial value, indicating that the state-retention time of our device is around 40 min. The long-term relaxation of G is also consistent with the hysteric proton permeation to the CaF_2_/graphene interface, presenting as a small and broad negative j peak over the long-term relaxation. Such consistency in the memristive changes of conductance state with the memristive change of $\sigma_{imp}$ and j (Fig. 3 *D* and *E*) verifies that aqueous protons regulate the conductance state of our memristive device. Corresponding molecular pictures are schematically depicted in Fig. 3*B*. Markedly, the similarities between our memristive device and synapses allow us to emulate the synaptic plasticity and the time-correlated learning process of Hebbian plasticity. Detailed results for long-term potential and depression, operation voltages, and ON/OFF time, as well as spike-time-depended-plasticity (STDP), can be found in *SI Appendix*, sections S13–S17.

The plasticity and state-retention reported here are not competitive with state-of-the-art memristive devices. Yet, the simplicity of our device, which can be relatively easily scaled down and multiplexed, and the perspectives for further improvements in the device performance provided by the molecular-level insights highlight the potential of 2D materials for aqueous memristive devices.

We implemented memristive behavior in aqueous electrolytes using monolayer graphene supported on a CaF_2_ substrate as a model system and probed in situ the ion dynamics of the neuromorphic devices with surface-specific vibrational spectroscopy. Our work demonstrates a unique and simple concept for developing aqueous electrolyte-based neuromorphic iontronics using two-dimensional (2D) materials through manipulating interfacial acid-base equilibrium. Our molecular-level insights into the underlying mechanism of the memristive behavior reveal that the graphene acts both as the conductivity modifier and reporter. It modifies the conductivity by acting as an electrochemical electrode, changing the pH, and itself as a pH sensor, by reporting the conductivity modifications. These insights guide developing 2D-material-based neuromorphic iontronics devices operating in aqueous electrolytes using protons as charge carriers. Furthermore, the memristive effect shows no dependence on the size of the graphene, demonstrating its potential for large-scale integration and multiplication. The method and findings presented here pave the way for resolving molecular-level details of the ion-mediated synaptic behavior in aqueous electrolyte-based neuromorphic iontronics and developing the next-generation brain-machine interfaces.

## Materials and Methods

Our aqueous proton-based memristive devices are simply 2D materials supported on a CaF_2_ substrate, contacted with two gold electrodes. In this work, we investigate several memristive devices, of similar geometry but of a different number of graphene layers (*n* = 1 to 2), different 2D materials [graphene and hexagonal boron nitride (hBN) purchased from Grolltex Inc.], and different substrates (SiO_2_ and CaF_2_). The schematic diagram of a prototype memristive device made by monolayer graphene and a CaF_2_ substrate is shown in Fig. 1*A*. Further details regarding the preparation of the devices can be found in references (49, 76, 77), and recalled in *SI Appendix*, *Methods*. Once prepared, the sample was mounted on a homemade electrochemical flowing cell. The electrochemical flowing cell enables us to conduct the electrochemical and conductance measurements together with Raman or HD-SFG measurement at the same time (*SI Appendix*, *Methods*). Since flow can affect the proton permeation through graphene, it can affect the memory effect in our memristive device. The higher the flow rates, the weaker the memory effect (*SI Appendix*, section S13). In this work, to avoid the change of bulk pH of the NaClO_4_ aqueous solution during our long-term electrochemical measurements and to best illustrate the memristive behavior, the electrolyte solution was flown through the cell at a constant flow rate (50 µm/s, unless stated otherwise) defined as the measured water volume flowing per unit of time divided by the cross-section of the flow channel. The flow rate was controlled using a variable flow syringe pump. For the HD-SFG measurement, we used 10 mM and 100 mM NaClO_4_, while we used 10 mM NaClO_4_ for Raman measurements. We have conducted control experiments using NaCl as the electrolyte, and all reported results were similar. The solution was purged with argon gas for at least 30 min before being used. To prevent oxygen leakage into the cell and to avoid spectral distortion due to water vapor, the HD-SFG spectra were measured in an N_2_ atmosphere. The measurements were performed at the *ssp* polarization combination, where *ssp* stands for *s*-polarized SFG, *s*-polarized visible, and *p*-polarized IR beams. More details about the graphene transfer and characterization, electrode preparation, and electrochemical cell design can be found in Supplementary methods.

## Supplementary Material

Appendix 01 (PDF)

## Data Availability

All study data are included in the article and/or *SI Appendix*.
