# Supplementary material for: Aqueous chemimemristor based on proton-permeable graphene membranes
Source: Proc Natl Acad Sci U S A. 2024 Feb 1;121(6):e2314347121. doi: 10.1073/pnas.2314347121 (PMC10861866; doi:10.1073/pnas.2314347121)
Supplement: Supplementary file 1 — Appendix 01 (PDF) [file pnas.2314347121.sapp.pdf]

Supporting Information for
Aqueous Chemimemristor Based on Proton-permeable Graphene Membranes.

Yongkang Wang,[1,2] Takakazu Seki,[2] Paschalis Gkoupidenis,[2] Yunfei Chen,[1*] Yuki Nagata,[2*] and Mischa Bonn[2*]

Yunfei Chen, Yuki Nagata, and Mischa Bonn
Email: yunfeichen@seu.edu.cn, nagata@mpip-mainz.mpg.de, bonn@mpip-mainz.mpg.de

This PDF file includes:

Supporting text
Figures S1 to S18

Supporting Information Text

Supplementary Methods

1.Chemicals.

All related chemicals of sodium hydroxide (NaOH), hydrochloride (HCl), concentrated sulfuric acid (H_2SO_4), 30 wt. % hydrogen peroxide solution (H_2O_2), ammonium persulfate ($(NH_4)_2S_2O_8$), cellulose acetate butyrate (CAB), ethyl acetate, ethanol, and acetone were purchased from Sigma-Aldrich and used as received. Sodium perchlorate (metals basis, 99.99%) was obtained from Merck, and used as an electrolyte solution in our electrochemical experiment with concentrations of 10 mM or 100 mM. Deionized water was provided by a Milli-Q system (resistivity ≥ 18.2 MΩ·cm and TOC ≤ 4 ppb) and was saturated with argon by bubbling gas through it for 30 minutes before use. CVD-grown monolayer graphene and hBN on copper foils were purchased from Grolltex Inc.

2.CaF_2/SiO_2 Substrate Preparation.

CaF_2 and SiO_2 windows (25 mm diameter with a thickness of 2 mm, PI-KEM Ltd.) were cleaned with acetone, ethanol, and deionized water sequentially in the ultrasonic environment for five minutes. Subsequently, the CaF_2 windows were immersed in an HCl solution at pH 2 for 2 hours to generate the fresh CaF_2 surface, while the SiO_2 windows were immersed in a piranha solution for 15 minutes before use. After that, two 100 nm-thick gold strips were thermally evaporated onto the CaF_2 and SiO_2 windows with a shadow mask. The gold strips enable us to measure the conductance of the graphene electrode and manipulate electrochemical potentials between the graphene and a reference electrode. Besides, the gold strips also serve as the reference sample to provide a stable and precise reference phase.

3.Graphene/hBN Wetting Transfer.

The CVD-grown monolayer graphene was transferred onto the cleaned CaF_2/SiO_2 windows by using the polymer-assisted wet transfer technique.(1) In brief, the copper foil was spin-coated with CAB at 1,000 rpm for 10 seconds, followed by 4,000 rpm for 60 seconds, and then baked at 180 °C for 3 minutes. After cooling down to room temperature, the copper foil/CAB was placed into HCl/H_2O_2/H_2O mixture solution (volume ratio, 1:1:10) for 60 seconds to remove the graphene layer grown on the backside of the copper foil. After being rinsed with deionized water, the copper foil on the film was then etched away in 0.1 M ammonium persulfate aqueous solution. Subsequently, the obtained CAB-graphene films were rinsed in deionized water several times to remove residual chemical species, and then were transferred onto CaF_2 and SiO_2 windows. The samples were dried for more than 12 hours at 110 ℃ in a vacuum (~1 mbar) to remove residual water. Finally, the CAB layer on graphene was dissolved in acetone. The monolayer hBN was transferred following the same procedures. Multilayer graphene samples were prepared by multistep transfer of monolayer. To ensure electrical connection, two gold wires were bound to the two gold strips on the CaF_2 and SiO_2 windows with conductive silver paste (TED PELLA, INC.).

4.Electrochemical Flowing Cell.

Our electrochemical flowing liquid cell is schematically depicted in Fig. S1. The cell mainly consists of two rectangular polytetrafluoroethylenes (PTFE) parts, the top clamp part, and the bottom flowing channel (12×3×3 mm^3) part. The top clamp has an opening of ~16 mm in diameter for the light beam paths. The bottom part has four round holes on the four side walls. Two for inlets and outlets of electrolyte solution (10mM or 100 mM $NaClO_4$) while another two for insertion of the hydrogen-loaded palladium wire (RE) and the gold wire (CE). The monolayer graphene electrode on a CaF_2 or a SiO_2 window and an O-ring were then sandwiched between the top and the bottom PTFE parts. The O-ring was used to create a seal between the electrolyte solution and the graphene electrode to avoid contact between the solution and the two gold strips. The base and clamp parts were cleaned with piranha solution before use.

5.Electrochemical Measurement.

A schematic diagram of the experimental setup for the electrochemical measurement is shown in Fig. S1. We used a three-electrode setup for the potential control. In this setup, the graphene

electrode serves as the WE. As the CE and RE, gold, and Pd/H_2 wires were used. These three electrodes were connected to an electrochemical workstation (Metrohm Autolab PGSTAT302). We use AC voltage of various frequencies (0.1 to 100 mHz) with a triangular waveform. The sampling rate is 0.1 Hz for the electrochemical, conductance, Raman, and HD-SFG measurements. All the measurements were repeated more than five times to improve the signal-to-noise ratio.

6.Conductance Measurement.
For the conductance measurement, two gold strips were connected to the Keithley Source Meter (SMU 2450), serving as the drain and source electrode, respectively. We conducted all the conductance measurements at the same source voltage (50 mV).

7.Raman Measurement.
The Raman spectra were recorded with a WITec confocal Raman spectrometer (alpha 300 R, ×10 objective) with 600 grooves/mm grating, 532 nm laser, 2 mW power, and 10 s integration time.

8.HD-SFG Measurement.
HD-SFG measurements were performed on a non-collinear beam geometry with a Ti:Sapphire regenerative amplifier laser system (Spitfire Ace, Spectra-Physics, centered at 800 nm, ~40 fs pulse duration, 5 mJ pulse energy, 1 kHz repetition rate). A part of the output was directed to a grating-cylindrical lens pulse shaper to produce a narrowband visible pulse (10 μJ pulse energy, FWHM = ~10 cm^{-1}), while the other part was used to generate a broadband infrared (IR) pulse (3.5 μJ pulse energy, FWHM = ~530 cm^{-1}) through an optical parametric amplifier (Light Conversion TOPAS-C) with a silver gallium disulfide ($AgGaS_2$) crystal. The IR and visible beams were firstly focused into a 200 nm-thick ZnO on a 1 mm-thick CaF_2 window to generate a local oscillator (LO) signal similar to the reference.(2) Then these beams were re-focused by two off-axis parabolic mirrors pair and overlapped spatially and temporally at the graphene/water interface. A fused silica glass plate with a 1.5 mm thickness was placed in the optical path for the LO signal between the two off-axis parabolic mirrors, allowing the phase modulation for the LO signal. The SFG signal from the sample interfered with the SFG signal from the LO, generating the SFG interferogram, which was then dispersed in a spectrometer (Shamrock 303i, Andor Technology) and detected by an EMCCD camera (Newton, Andor Technology). HD-SFG spectra were measured in an N_2 atmosphere to avoid spectral distortion due to water vapor. To avoid height change of the sample surface upon flowing electrolyte solutions, we used a height displacement sensor (CL-3000, Keyence). Each spectrum was acquired with an exposure time of 10 seconds, and measured more than ten times on average. IR, visible, and LO beams were re-focused onto the graphene/water interface at the angles of incidence of 33°, 39°, and 37.6°, respectively. The measurements were performed at the *ssp* polarization combination, where *ssp* denotes *s*-polarized SFG, *s*-polarized visible, and *p*-polarized IR beams. The complex-valued spectra of second-order nonlinear susceptibility ($\chi^{(2)}$) of the graphene/water interface samples were obtained via the Fourier analysis of the interferogram and normalization with that of the CaF_2/gold interface. The interferogram of the CaF_2/gold interface was collected at the gold strip region of the sample immediately before the sample measurement to ensure a precise and stable reference phase. The phase of the thin gold film is determined by measuring the O-H stretching $\mathrm{Im}(\chi^{(2)})$ spectrum of the CaF_2-supported graphene/D_2O interface via normalization of the signal with that of CaF_2/gold. As D_2O does not have any vibrational response in this region, and its $\chi^{(2)}$ response arises solely from the interface,(3) we can determine the phase of gold based on the fact that the $\mathrm{Im}(\chi^{(2)})$ spectrum of the CaF_2-supported graphene/D_2O interface shows a flat zero line.

Supplementary discussion

S1. Characterization of the Monolayer Graphene.

An optical image of the transferred monolayer graphene on a CaF_2 substrate is shown in the inset in Fig. S2. The optical image shows that the graphene electrode is complete and clean. Two gold strips were thermally deposited onto the CaF_2 window before the graphene transfer for electrical connection and conductance measurement. Besides, the gold strips also serve as the reference sample to provide a stable and precise reference phase.

We also measured the Raman spectra of the graphene electrode before and after the electrochemical (EC) measurements (Fig. S2a). The two Raman spectra show no changes and the absence of the defect-related D-band, demonstrating that the graphene remains electrochemically intact within the present electrochemical window.(4, 5)

Defects are unavoidable during the CVD synthesis and transfer processes. Indeed, previous works has shown that the density of defects is around 10^{10} cm^{-2} (100 defects per μm^2)(6) even though the absence of a Raman D-band. As device integration moves towards the nanoscale, defects and their distribution are expected to impact the device performance and device-to-device variability. Nevertheless, given that our devices have a dimension ranging from ~70×500 μm^2 to ~1×2 cm^2, the distribution of defects on the graphene should be regarded as rather uniform and therefore, we concluded that the random distribution of defects in graphene did not impact the performance of our aqueous proton-based memristive device. (see also the highly reproducible memristive conductance change in Fig. S2b).

To further verify this notion, we fabricated the memristive device using CaF_2-supported defective graphene and measured graphene conductance as a function of V_g. The defective graphene device was fabricated by treating the CaF_2-supported graphene with UV Ozone for 3 minutes.(7) The Raman D to G peak intensity ratio indicates that a density of ~5×10^{11} cm^{-2} defects(8) were induced on the graphene after the UV Ozone treatment (Fig. S2c). The overall conductance for the defective graphene device is lower because of an overall low mobility with its higher defect density.(9) Still, the $G - V_g$ curve displays significant memristive behavior (Fig. S2d). Consequently, we concluded that the random distribution of defects in graphene did not impact the performance of our aqueous proton-based memristive device.

S2. The Memristive Effect at Various AC Frequency.

We measured $G - V_g$ curves at various AC frequencies of the applied gate potentials. The data is shown in Fig. S3. The memristive effect was found to take place at frequencies between 0.17 and 17 mHz, corresponding to memory timescales from tens of seconds (~60s) to thousands of seconds (~6000s). The memory effect is weakened at high frequency because the extent of discharging of CaF_2 in backward scan decreases. This can be inferred from the left shift of the crossing point in the $G - V_g$ curves. The weakened discharging of CaF_2 at high frequency is because the local pH change-induced discharging of CaF_2 occurs on the scale of seconds.(10) At high frequencies, discharging of CaF_2 (I) is not faster enough to follow the change of V_g. The higher the frequency, the smaller the extent of the discharging of CaF_2 and the weaker the memory effect.

S3. Micrometer-sized Graphene Memristive Device.

To show that the aqueous electrolyte-based graphene memristive device can be scaled down for large-scale integration and multiplication, we made the memristive device using graphene micro-stripes. The graphene micro-stripes were prepared using the oxygen-plasma etching method. In brief, we first coated gold micro-stripes onto the CaF_2-supported monolayer graphene using a mask (Fig. S4a). The gold micro-stripes serve as a protective layer and the graphene sample was etched by oxygen-plasma (300 W, 5 minutes). Then the gold micro-stripes were etched using KI gold etchant and finally, the sample was rinsed with deionized water. An optical image of the obtained graphene micro-stripes on the CaF_2 substrate is shown in Fig. S4b. We measured the $G - V_g$ curve using the micrometer-sized graphene memristive device and the data is shown in Fig. S4c. The $G - V_g$ curve exhibits a significant memory effect, indicating that the memory effect in our graphene

memristive device doesn't show dependence on the size of the graphene. These results also suggest that our graphene memristive devices allow for large-scale integration and multiplication.

S4. Minimum Conductance Measurement.

Due to the charged impurity scattering, the minimum conductance of the graphene may also change.(9) Accordingly, we measure the G_{min} as a function of the time-varying V_{g}. The data is shown in Fig. S5. The G_{min} change is small and negligible, which is plausible because the variation of σ_{s} is not significant. For simplicity, we use a constant G_{min} to calculate the μ_{G}.

S5. Charge Carrier Density on Graphene.

We use Raman spectroscopy to independently determine the graphene Fermi level, and thereby its charge carrier density (n_{g}) from the Raman G-band frequency shift.(11, 12) The Fermi level in graphene (E_F) changes with the density of the charge carriers via $E_{\mathrm{F}} = \hbar|v_{\mathrm{F}}|\sqrt{\pi n_{\mathrm{g}}}$, where $v_{\mathrm{F}}(= 1.1 \times 10^{-6}\ \mathrm{ms}^{-1})$ is the Fermi velocity in monolayer graphene,(13) and n_{g} denotes the charge carrier density in graphene. Meanwhile, it has been shown that the G-band Raman frequency shifts linearly as a function of the Fermi level, $E_{\mathrm{F}} = 21\Delta\omega_{\mathrm{g}} + 75\ [\mathrm{cm}^{-1}]$ for electrons and $E_{\mathrm{F}} = -18\Delta\omega_{\mathrm{g}} - 83\ [\mathrm{cm}^{-1}]$ for holes. Surface charge density on graphene at various electrochemistry potentials can therefore be determined via Raman spectral analysis of the G-band Raman frequency shifts. The Raman spectra of the graphene electrode when changing the applied potentials are shown in Fig. 1c, corresponding n_{g} as a function of V_{g} is shown in Fig. 1d.

In addition to the charge carrier density, other factors such as mechanical strain(14) and ion absorption(15, 16) on the graphene could also induce the Raman G-band frequency shift. The first one is inevitable for supported monolayer graphene. In our sample, the induced frequency shift is around 2 cm^{-1}. We estimate this value from the Raman G-band frequency at the charge-neutral point (1584 cm^{-1} at 0.1 V, Fig. 1c), considering that the G-band frequency of freestanding graphene is 1582 cm^{-1}. Since the mechanical strain does not affect the effect of charge carrier density on the G-band frequency shift.(14) we treat it as a constant value and subtract it when calculating the charge carrier density.

To rule out the ion absorption effect on the Raman G-band frequency shift, we measured the Raman spectra of the graphene electrode at different pH conditions at the OCP condition. The data is shown in Fig. S6. Changing pH from 7 to 11 does not affect the G-band frequency shift indicating that the observed Raman G-band frequency shift upon applying V_{g} is hardly affected by the ion absorption on the graphene surface.

S6. Pseudocapacitive Charging/discharging of CaF_2.

That the σ_{imp} changes upon the variation of V_{g} is very plausible and has been demonstrated in our recent work,(10) which is attributed to the local pH change-induced pseudocapacitive charging/discharging of the CaF_2 substrate. The CaF_2 substrate is positively charged at neutral pH (~7), with its isoelectric point in the pH range of 9 to 10.(17, 18) Applying negative potentials, interfacial water dissociation occurs, and subsequent proton permeation through the graphene electrode raises the local pH near the graphene electrode. The raised local pH induces the adsorption of hydroxide ions on the CaF_2 surface and, thus, the discharge of the CaF_2.

To verify this, we measured cyclic voltammogram (CV) curves on the graphene electrode. The CV curves show the water dissociation current at a potential below -0.3 V (Fig. S7a). Importantly, a hysteresis loop in the CV curve is observed. The water dissociation is accompanied by proton permeation and can be correlated with the proton permeation through the graphene electrode.(19) This self-crossed hysteresis loop indicates a hysteretic proton permeation through the graphene electrode, consistent with the conductance measurement and HD-SFG measurement that the proton permeation exhibits long-term memory and synapse-like dynamics.

The distribution of water trapped between graphene and CaF_2 substrate is an important factor because it affects the charging/discharging of the CaF_2 surface (σ_{imp}, see chemical equilibrium of (I) in the Main text). In our device, the water trapped between graphene and CaF_2 substrate is sub-monolayer and appears to be distributed uniformly, as apparent from topographic AFM study in our previous study.(10) Based on this observation, we conclude that the variability in the distribution of the trapped water is negligibly small. This thin water layer, enables the operation of the device by facilitating proton transfer, but does not affect the performance of our aqueous proton-based memristive device.

To further verify this notion, we measured the HD-SFG spectra at the CaF_2-supported graphene/water interface at different sample positions and from different samples. For each measurement, HD-SFG spectroscopy probes a spot of ~100 μm. Within experimental error, the $\mathrm{Im}(\chi^{(2)})$ spectra exhibit negligible variations (Fig. S7b), indicating that the distribution of water trapped between graphene and CaF_2 substrate is uniform over the beam spot with its diameter of ~100 μm. Likewise, the memristive conductance change was highly reproducible (Fig. S2b). Therefore, we concluded that the wetting transfer approach does not cause the variability of water amounts in the device and our device demonstration is rather robust.

S7. Extraction of σ_{imp} from $\chi^{(2)}$ Spectra.

The PA of the H-bonded O-H band is proportional to the density of charge impurities because the variations of the $\chi^{(2)}$ spectra (10 mM ions concentration) mainly come from the bulk contribution, i.e., the $\chi^{(3)}$ contribution.(20) To obtain the linear dependence, we first obtain σ_{imp} at +0.3 V and -1.1 V, following the procedures developed by Shen et al. and Bonn et al.(21, 22) In brief, we measured the differential spectra $\Delta\chi^{(2)}(\sigma_{\mathrm{imp}}, V_{\mathrm{g}}) = \chi^{(2)}(\sigma_{\mathrm{imp}}(V_{\mathrm{g}}), c_1 = 10\ \mathrm{mM}) - \chi^{(2)}(\sigma_{\mathrm{imp}}(V_{\mathrm{g}}), c_2 = 100\ \mathrm{mM})$ and divided the $\Delta\chi^{(2)}$ with $\chi^{(3)}$ (third-order nonlinear susceptibility originating from bulk water and is constant). σ_{imp} can then be obtained from the comparison of the experimentally obtained left side and the computed right side of Eq. S1 basing on the Gouy-Chapman theory.

$$\frac{\Delta\chi^{(2)}(\sigma_{\mathrm{imp}}(V_{\mathrm{g}}), c_1, c_2)}{\chi^{(3)}} = \frac{\phi_0(\sigma_{\mathrm{imp}}, c_1)\kappa(c_1)}{\kappa(c_1) - i\Delta k_z} - \frac{\phi_0(\sigma_{\mathrm{imp}}, c_2)\kappa(c_2)}{\kappa(c_2) - i\Delta k_z}, \tag{S1}$$

where $\phi_0 = \frac{2k_BT}{e} sinh^{-1}\left(\frac{\sigma_{\mathrm{imp}}}{\sqrt{8000\varepsilon_0\varepsilon_r k_B T N_A c}}\right)$ is the electrostatic potential, $\kappa = \sqrt{\frac{2000e^2N_Ac}{\varepsilon_0\varepsilon_r k_BT}}$ is the inverse of Debye screening length, c is the electrolyte concentration and Δk_z is the phase-mismatch of the SF, visible, and IR beams in the depth direction. The obtained $\Delta\chi^{(2)}$ spectra at +0.3 V and -1.1 V are shown in Fig. S8 and the extracted σ_{imp} is +44 mC/m^2 and -6 mC/m^2 respectively. Considering that the PA is -4.58 and -0.48, we obtain $\sigma_{\mathrm{imp}} = -(\mathrm{PA} + 0.97)/0.082$. we note the intercept of 0.97 represents the contribution from surface water to the $\mathrm{Im}(\chi^{(2)})$ spectra.

S8. Correspondence of μ_{g} and PA.

Due to the long-range Coulomb scattering, μ_{g} is expected to be proportional to the inverse of the density of charge impurities (σ_{imp}).(9) Ionic screening of the charge impurities is smaller at 10 mM ion concentration and is ignored in this work.(23, 24) Indeed, the variation of μ_{g} at various V_{g} show good consistency with the variation of $1/\sigma_{\mathrm{imp}}$ inferred from PA, as seen in Fig. S8. The correspondence verifies that the memristive charging/discharging of the CaF_2 substrate is responsible for the memory effect in our aqueous proton-based memristive device. We note that $\mu_{\mathrm{g}} \propto 1/\sigma_{\mathrm{imp}}$ is an empirical formula. It provides good description at $\sigma_{\mathrm{imp}} > 1.6$ mC/m^2 (carrier density of ~10^{12} cm^{-2})(9) but is inaccurate when σ_{imp} approaches zero which gives rise to an infinite μ_{g} (Fig. S9). An accurate description of the relationship between μ_{g} and σ_{imp} will help but is beyond the scope of this study.

S9. Reproducibility.

To demonstrate the reproducibility of our aqueous proton-based memristive device, we measured the $\mathrm{Im}(\chi^{(2)})$ spectra when applying a series of periodic V_g pulses. The obtained PA of the H-bonded O-H band is shown in Fig. S10. The variation of PA is highly reproducible, demonstrating that the memristive charging/discharging process at the CaF_2-supported graphene/water interface is highly reproducible.

S10. Negligible Ion-specific Effect.
We measured G of the CaF_2-supported graphene electrode in contact with 10 mM NaCl aqueous solution (pH ~7) when probed by time-varying V_g. The data is shown in Fig. S11. A similar memristive effect compared to that using 10 mM $NaClO_4$ is observed, indicating negligible ion-specific effects in our aqueous proton-based memristive device.

S11. Memristive Devices Using Other 2D Materials.
We further fabricated the aqueous proton-based memristive devices using CaF_2-supported double-layer graphene, CaF_2-supported graphene/hBN heterostructure, and SiO_2-supported monolayer graphene. To demonstrate their ability to exhibit the memory effect, we measured the $\mathrm{Im}(\chi^{(2)})$ spectra when applying the time-varying V_g. The data is shown in Fig. S12. The memory effect is remarkable in all three devices. The demonstration using CaF_2-supported graphene/hBN heterostructure indicates that any membranes allowing for proton permeation can be used to fabricate the aqueous proton-based memristive devices.

For SiO_2-supported monolayer graphene, PA is always positive and increases with lowering V_g. This is because the isoelectric point of SiO_2 is around pH 2 and SiO_2 is negatively charged at neutral pH (~7). Changing the potential from positive to negative increases the local pH. Surface charges on SiO_2 increase, explaining the increase of PA.(10)

The memory effect observed in the CaF_2-supported double-layer graphene suggests that energy barriers for protons to permeate through the graphene electrode cannot explain the observed memory effect. Otherwise, we would expect a significant increase in memory in the CaF_2-supported double-layer graphene device, which is not the case.

S12. Diffusion-limited Proton Permeation Through Graphene.
We estimate the net proton permeation current density (j) through graphene via;

$$j = \frac{d\sigma_{\mathrm{imp}}}{dt}. \qquad (S2)$$

The variations of j at different v_s are depicted in Fig. 2d. The $j - V_\mathrm{g}$ curve exhibits an asymmetric positive peak at around -0.7 V in backward scanning the potential. The positive peak means proton permeation from the CaF_2/graphene interface to the bulk water and the discharging of the CaF_2. In forward scanning, the peak changes its sign to negative and shifts its position to around -0.4 V. The negative value suggests reverse proton permeation from the bulk water to the CaF_2/graphene interface and, thus, the charging of the CaF_2. Importantly, the peak shift confirms the hysteretic proton permeation through the graphene. v_s does not affect the positive peak but significantly decreases the peak shift of the negative peak, verifying that the observed memory effects in our aqueous proton-based memristive device mainly arise from the hysteretic proton permeation from the bulk water to the CaF_2/graphene interface.

To verify the hypothesis that the memory effect arises from the diffusion-limited proton permeation, we measured the $\mathrm{Im}(\chi^{(2)})$ spectra at different flow rates. The calculated j is shown in Fig. S13a. The negative peak of the proton permeation current shifts down towards negative potential with increasing the flow rate. This is because the faster bulk supply rate of protons (convection) by increasing flow rate disrupts the interface and weakens the memory effect. This hypothesis is also supported by the increased memory upon an increase of ion concentration (Fig. S13b).(25)

S13. Potentiation and Depression.
Applying a negative spike causes the discharge of the CaF_2, which raises μ_G and increases G (bottom panel in Fig. S14a.). Because the charging/discharging of CaF_2 can also change the Dirac point of the graphene and graphene's conductance is bipolar, the $G - V_g$ relationship near the Dirac point should be similar to the sketch in Fig. S14b. The graphene conductance change can therefore be below or above its initial state when the memristive device is operating at a potential lower or higher than the Dirac point (Fig. S14a). This suggests that our memristive device allows for switching between two typical forms of synaptic plasticity: long-term potentiation (LTP) and long-term depression (LTD).(26)

S14. Memory Phenomenon.
The long-term plasticity enables the storage of information through the synapse's conductance state. As a proof of concept, we implement the memory phenomena via incrementing and decrementing the G by applying 'write' (V_g = -0.4 V, 10 seconds) and 'erase' (V_g = 0.4 V, 10 seconds) voltage spikes respectively, see Figs. S15b and S15c. The data shows that the G states can be reversibly written and erased (potentiated and depressed) by applying a series of 'write' and 'erase' spikes. Importantly, this suggests that our aqueous proton-based memristive device allows for long-term, reversible, and programable modification and access to the stored value.

S15. Operating Voltage.
To evaluate the performance of our aqueous proton-based memristive device in terms of operating voltage, we measured the graphene conductance change as a function of V_g at different scan ranges towards negative potentials. The data is shown in Fig. S16. The $G - V_g$ curves display noticeable hysteretic changes even for an operating voltage low to 0.15 V, and the hysteretic changes increase with decreasing the operating voltage. We stress that an operating voltage of 0.15 V is a low and competitive voltage compared to most of the reported memory devices(27, 28) and is comparable to the biologically relevant voltage (bio-voltage, e.g., 50–150 mV).(28, 29) Thus, our concept and device carrier significance for both neuromorphic computing and brain-machine interface engineering. They are promising for realizing low-power consumption memristive devices.

S16. ON/OFF time.
We also characterized the ON/OFF time of our aqueous proton-based memristive device at different operating voltages (Fig. S17). The data shows that the ON/OFF time increases with lowering the operating voltage. This is because of more significant local pH changes at lower applied voltages which requires longer time to reset the system. The data indicates that our aqueous proton-based memristive device allows for long-term and voltage-tunable memory, demonstrating its potentials use for long-term, reversible information storage. We emphasis that a memory time of tens of minutes in our aqueous proton-based memristive device is not competitive with state-of-the-art solid-state memristive devices,(30, 31) but is privileged compared with state-of-the-art aqueous electrolyte-based memristive devices.(29, 32)

S17. Demonstration of STDP.
Considering the similarities between our memristive device and synapses, we demonstrate that our memristive device can be used to mimic the basic form of a Hebbian learning process. In biological neuron networks, this process involves modifying the synaptic weight depending on the relative activation time of two neurons connected by a given synapse.(33) The synapse is strengthened if the presynaptic neuron repeatedly emits an action potential just before the activation of the post-synaptic neuron, and is weakened if the firing order is reversed. Therefore, Hebbian learning is a mechanism that correlates the timing between pre- and post-synaptic input signals.

To mimic the Hebbian learning process, we applied a train of spikes and measured the conductance change. Similar to previous studies,(34) a time-division multiplexing approach is employed, which converts the difference in spike timing into various pulse amplitudes. First of all, we mimic the presynaptic activation and the post-synaptic activation using switching spikes V_{pre}

and V_{post}. V_{pre} and V_{post} have the same shape, but they generally arrive at two different times t_{pre} and t_{post} with the relative timing $\Delta t = t_{\mathrm{pre}} - t_{\mathrm{post}}$, as seen in Fig. S18. The voltage V_{g} across the device is $V_{\mathrm{g}} = V_{\mathrm{pre}} - V_{\mathrm{post}}$, which is illustrated in Fig. S18 for various values of Δt. Conductance change after a successive activation of the two neurons in the percentage of the initial conductance as a function of the relative activation timing is shown in Fig. S15d. The synapse is strengthened at $\Delta t > 0$ and is weakened at $\Delta t < 0$. This suggests the ability of our memristive device to mimic the Hebbian learning process, which is promising for time-correlated learning.

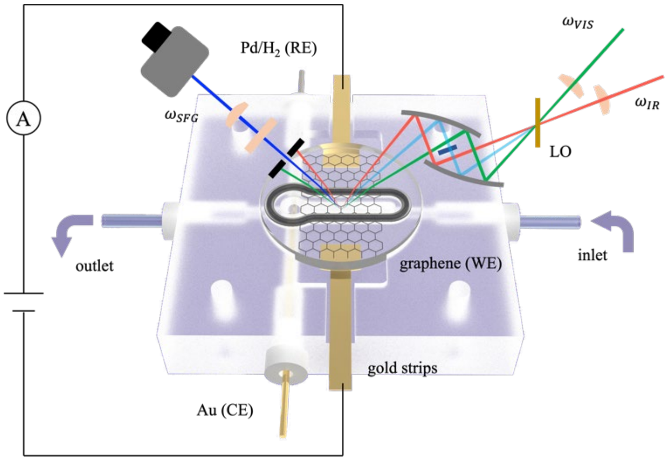

Fig. S1. Experimental setup. Schematic diagram of the electrochemical flowing liquid cell for conductance, Raman, and HD-SFG measurements. Graphene, Pd/H_2, and Au wire serve as the WE, RE, and CE, respectively. The two gold strips serve as the source and drain electrodes, respectively. The inset of the flow cell is connected to a variable flow syringe pump for flow rate control.

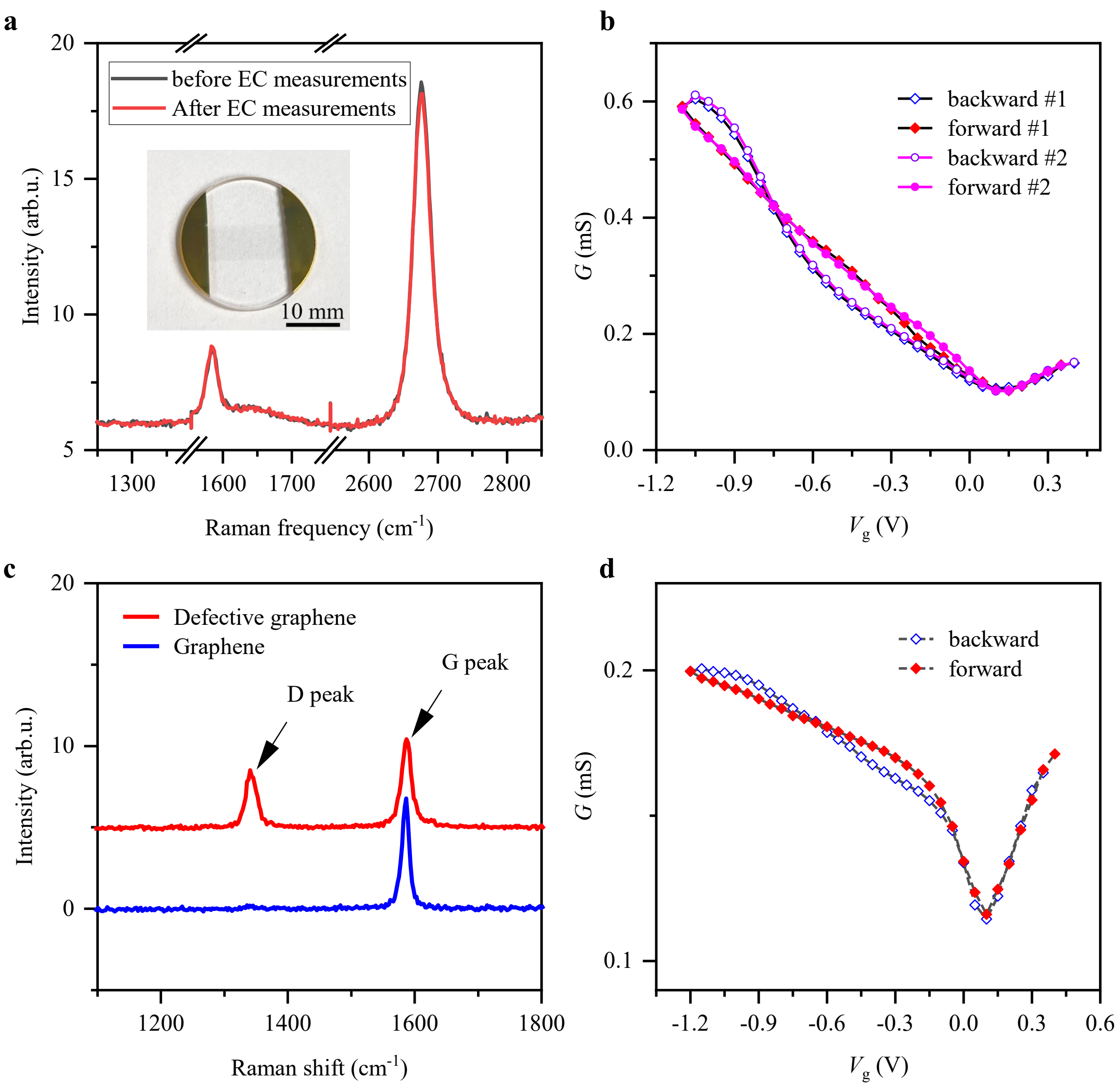

Fig. S2. Characterization of the monolayer graphene-based aqueous proton memristive device. **a**. Raman spectra of the graphene electrode at open circuit potential (OCP) condition, before and after the electrochemical measurements. The inset shows the optical image of the monolayer graphene-based memristive device. **b**. Memristive effect of different CaF_2-supported graphene devices. The open diamonds (circles) and the solid diamonds (circles) indicate the backward and forward scan, respectively. **c**. Raman spectra of the CaF_2-supported graphene (blue) before and (red) after UV Ozone treatment. **d**. $\boldsymbol{G}$ as a function of $\boldsymbol{V_g}$ for the aqueous proton-based memristive device made of CaF_2-supported defective graphene. The open blue diamonds and the solid red diamonds indicate the backward and forward scans, respectively. We used 10 mM $NaClO_4$ aqueous solution for **a-d** measurements. Measurements were conducted at a flow rate of 50 μm/s, a scan rate of 5 mV/s, and a step of 50 mV.

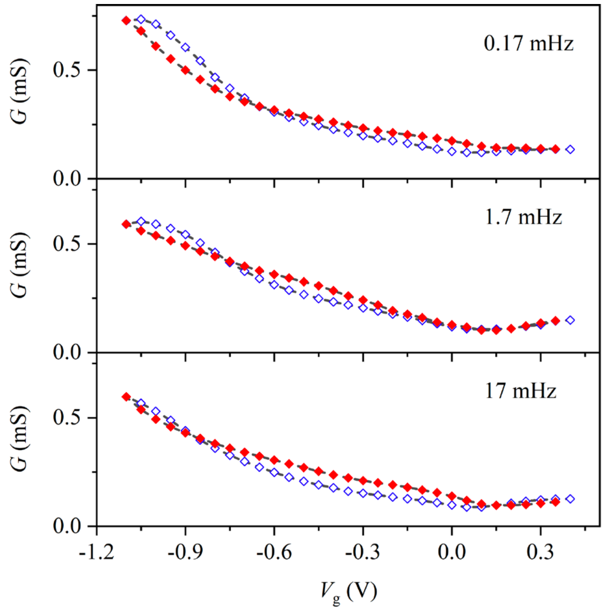

Fig. S3. G as a function of the time-varying V_g at various AC frequencies of V_g. We used 10 mM $NaClO_4$. The open blue diamonds and the solid red diamonds indicate the backward and forward scans, respectively.

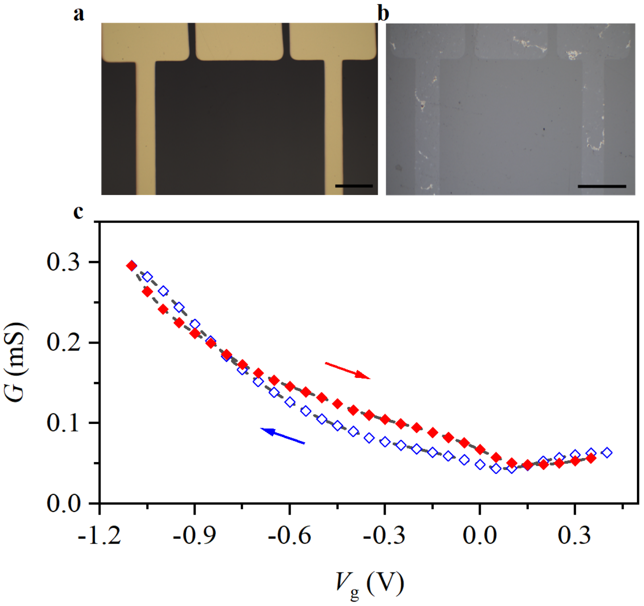

Fig. S4. Micrometer-sized graphene memristive device. **a**, **b**. optical images of the graphene memristive devices before and after gold etching. Scale bars indicate 150 *μ*m. **c**. $\boldsymbol{G}$ as a function of $\boldsymbol{V}_{\mathbf{g}}$. We used 10 mM $NaClO_4$. The open blue diamonds and the solid red diamonds indicate the backward and forward scans, respectively.

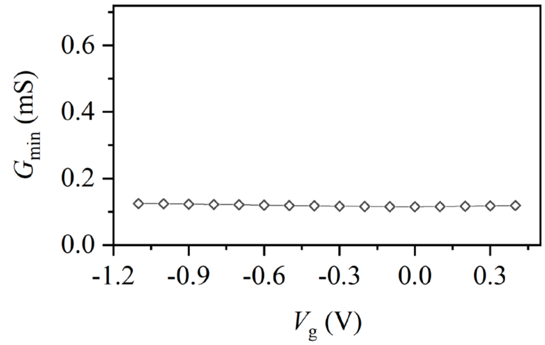

Fig. S5. G_{min} of the graphene electrode as a function of V_g. We used 10 mM $NaClO_4$. The G_{min} was measured within one second after each V_g pulse (10 seconds).

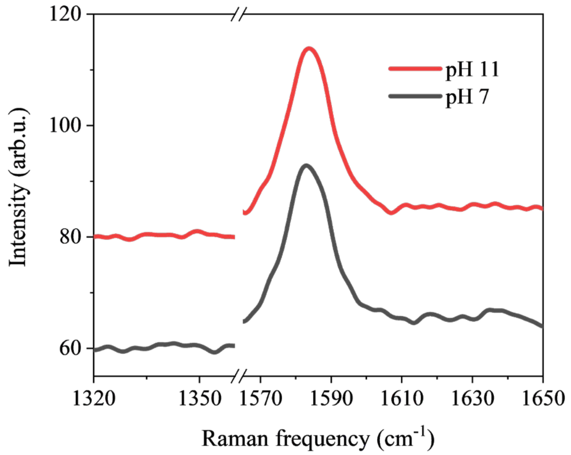

Fig. S6. Ruling out the ion absorption effect on the Raman G-band frequency shift. Raman spectra of the graphene electrode at pH 7 and pH 11 under open circuit potential (OCP) conditions. We used 10 mM $NaClO_4$.

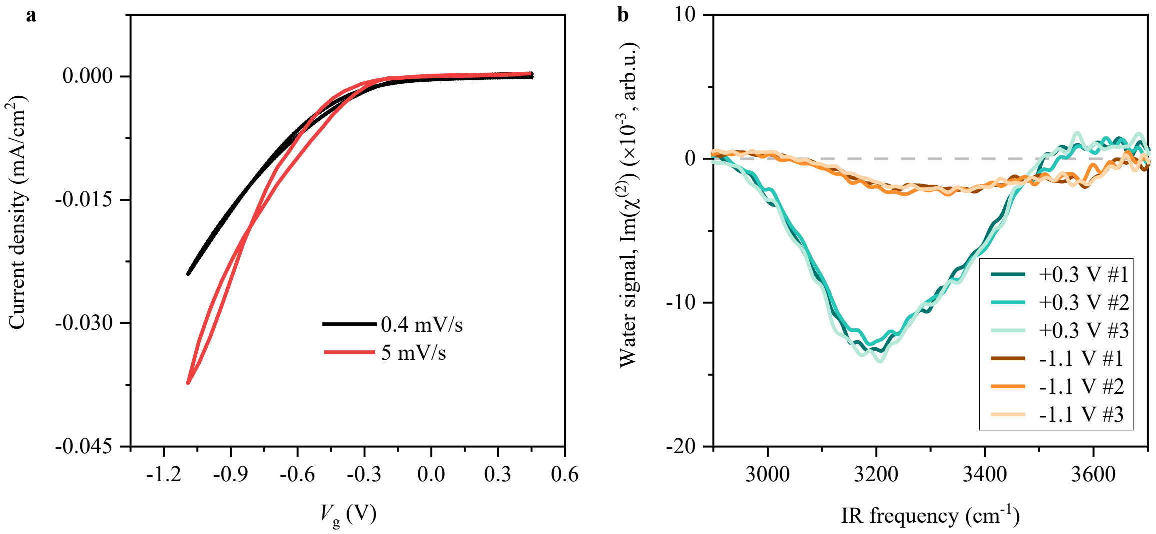

Fig. S7. Pseudocapacitive charging/discharging of CaF_2. a. Cyclic voltammogram of the graphene electrode at various v_s. **b.** The O-H stretching $\mathbf{Im}(\chi^{(2)})$ spectra at the CaF_2-supported graphene/water interface measured at different sample positions and from different samples, under different $\boldsymbol{V_g}$. Measurements were conducted at a flow rate of 50 μm/s. Signals #1 and #2 were measured at different sample positions on the same sample and signal #3 was measured on another sample. All the spectra were normalized by the CaF_2/gold signal. The dashed line indicates the zero line. We used 10 mM $NaClO_4$ aqueous solution for **a** and **b** measurements.

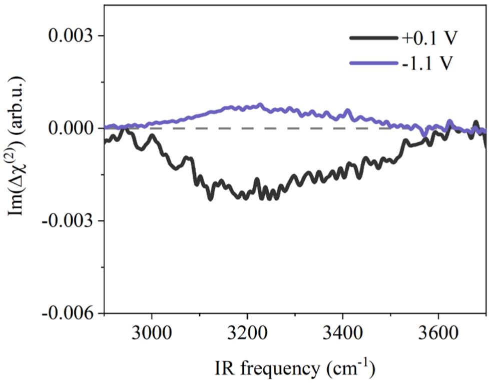

Fig. S8. The differential $\mathrm{Im}\left(\Delta\chi^{(2)}\right)$ spectra at +0.3 V and -1.1 V. The dashed line serves as the zero line.

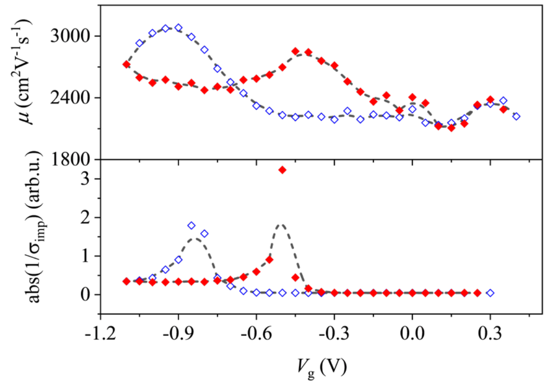

Fig. S9. Correspondence of $\boldsymbol{\mu_g}$ and $\boldsymbol{\sigma_{imp}}$. μ_g obtained from G and n_g show some small fluctuations near 0.1 V, the charge neutral point of the graphene, because n_g measured from Raman spectra is not accurate in this region. The open blue diamonds and the solid red diamonds indicate the backward and forward scan, respectively.

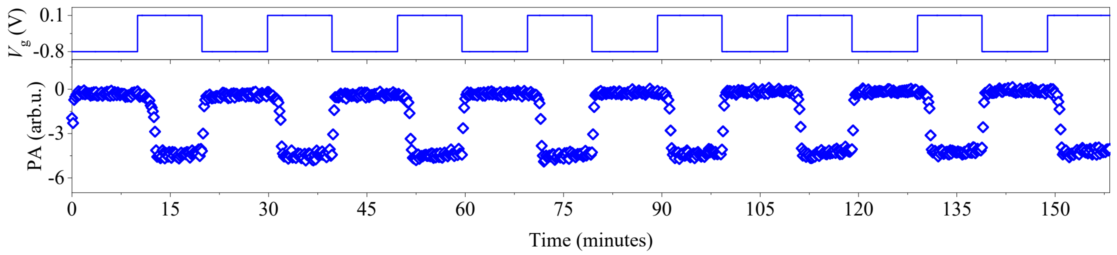

Fig. S10. Reproducibility of the memristive charging/discharging process at the CaF_2-supported graphene/water interface. We used 10 mM $NaClO_4$ solutions.

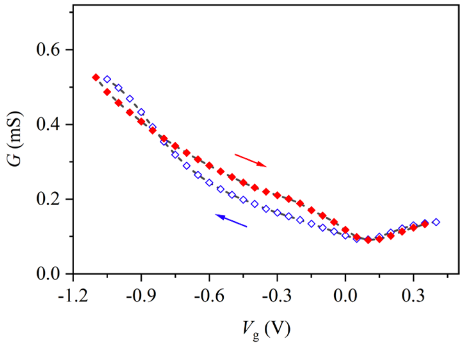

Fig. S11. Negligible ion-specific effect. G-V_g curve measured on the CaF_2-supported graphene electrode using 10 mM NaCl aqueous solution. The open blue diamonds and the solid red diamonds indicate the backward and forward scan, respectively.

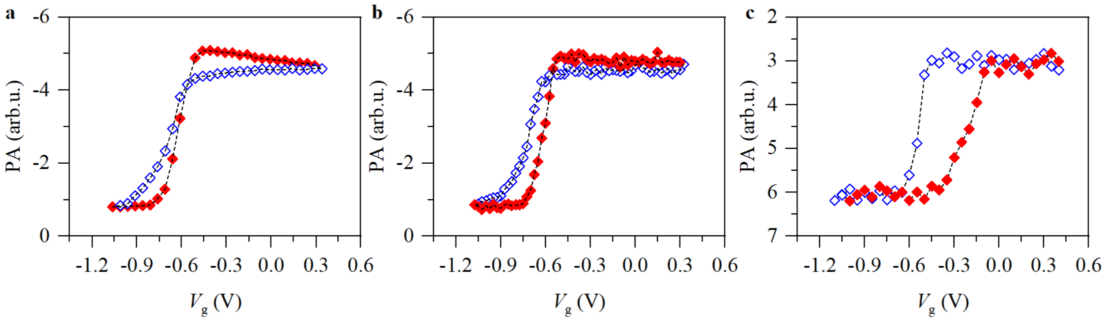

Fig. S12. PA as a function of the time-varying V_g using different memristive devices. The memristive device made by (**a**) CaF_2-supported double-layer graphene, (**b**) CaF_2-supported graphene/hBN heterostructure, and (**c**) SiO_2-supported monolayer graphene. We used 10 mM $NaClO_4$. The scan rate is 5 mV/s. The flow rate is 200 μm/s for **a** and **b** and 50 μm/s for **c**. The open blue diamonds and the solid red diamonds indicate the backward and forward scan, respectively.

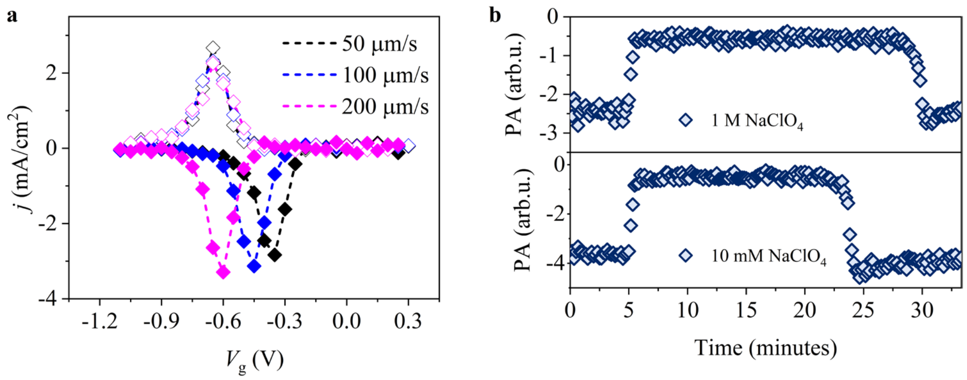

Fig. S13. Proton permeation through graphene at various flow rates. a. j as a function of V_g at various flow rates. The scan rate is 5 mV/s. We used 10 mM $NaClO_4$. The open and solid diamonds indicate the backward and forward scans, respectively. **b**. j as a function of time after a spike (-0.9 V) at different flow rates. We used 10 mM $NaClO_4$. The flow rate is 50 μm/s.

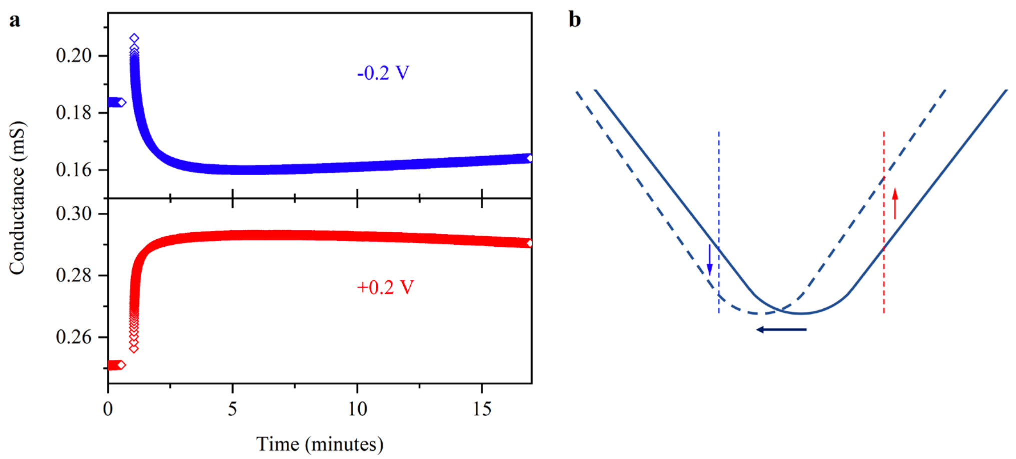

Fig. S14. Graphene conductance change probed at different operation gate potentials. a. Conductance changes at operation gate potentials of -0.2 V and +0.2 V after applying a -0.8 V spike (30 seconds). **b**. $G - V_g$ relationship change (dark blue line) before and (dashed dark blue line) after a 'write' spike. The dashed blue and red lines indicate the operation gate potential of -0.2 V and +0.2 V, respectively. We used 10 mM $NaClO_4$.

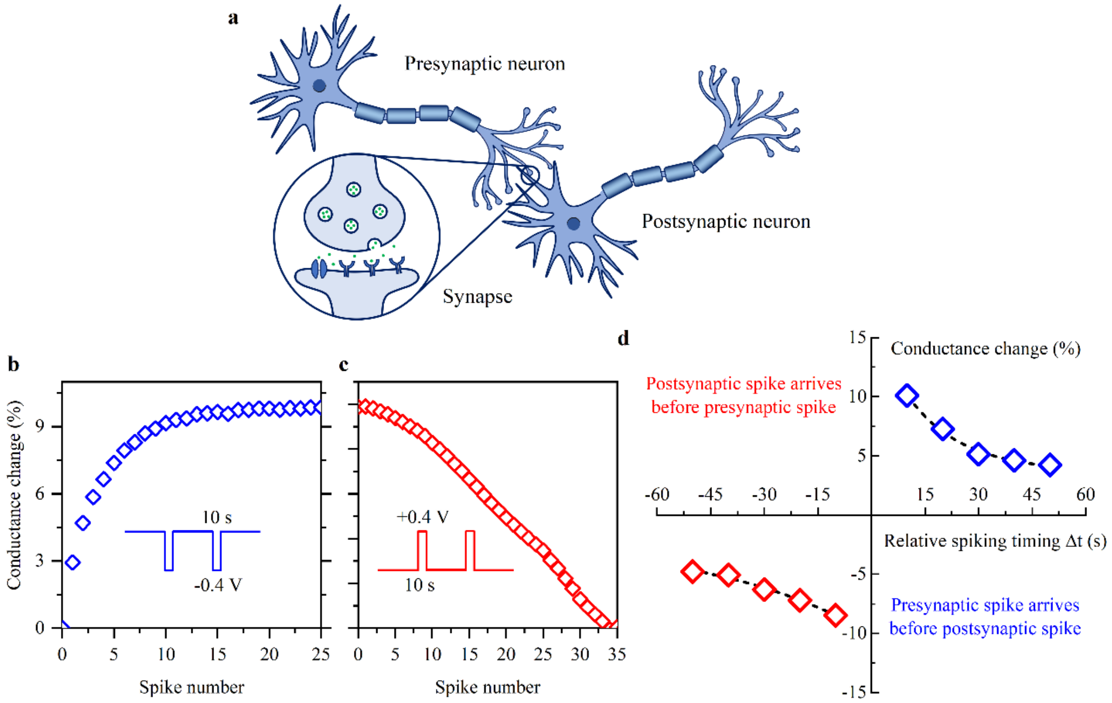

Fig. S15. Implementation of in-memory computation and Hebbian learning using the aqueous proton-based memristive device. a. Diagram of the biological neural network. The inset shows the single neuron synapse. Protons serve as the neurotransmitter (green dots) allowing for communication between the presynaptic neuron and the post-synaptic neuron. **b** and **c**. Modification of the graphene conductance via a series of 'write' and 'erase' spikes. The insets show the 'write' and 'erase' voltage spikes. The operation gate potential is 0.2 V. **d**. Conductance change relative to the initial conductance after a successive activation using two neuromimetic potentials, as a function of the relative activation timing.

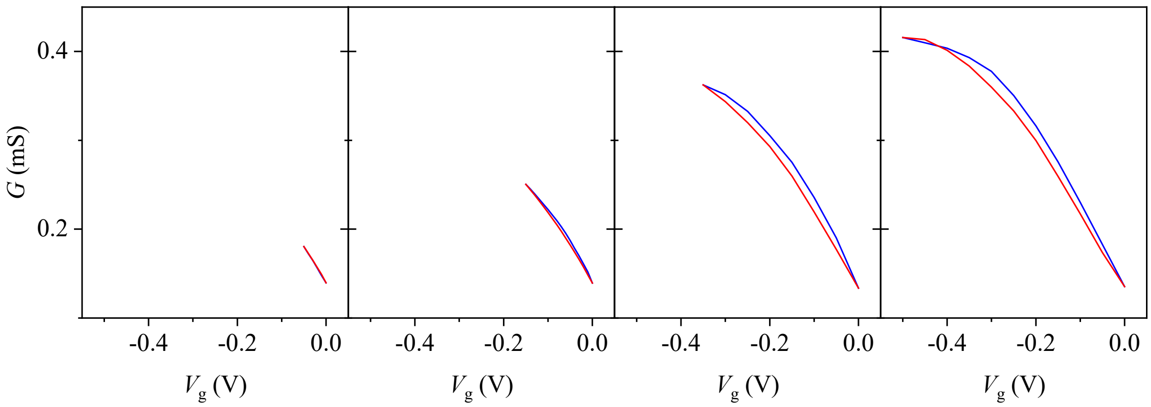

Fig. S16. Memristive graphene conductance change at different operation voltages. We used 10 mM $NaClO_4$ as the electrolyte. Measurements were conducted at a flow rate of 50μm/s and a scan rate of 5 mV/s. The blue lines and the red lines indicate the backward and forward scan, respectively.

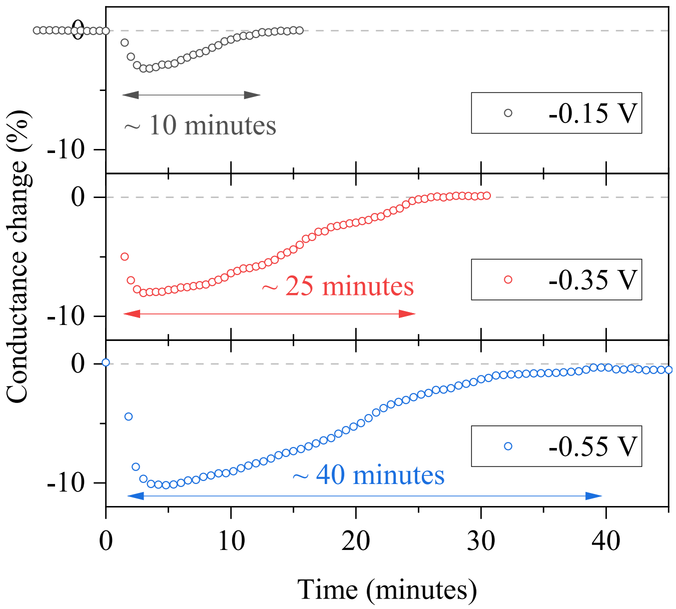

Fig. S17. ON/OFF time at different operation voltages. Graphene conductance changes as a function of time upon application of different 'write' spikes (30 seconds). We used 10 mM $NaClO_4$ as the electrolyte. Measurements were conducted at a flow rate of 50 μm/s. The dashed lines indicate the corresponding initial state.

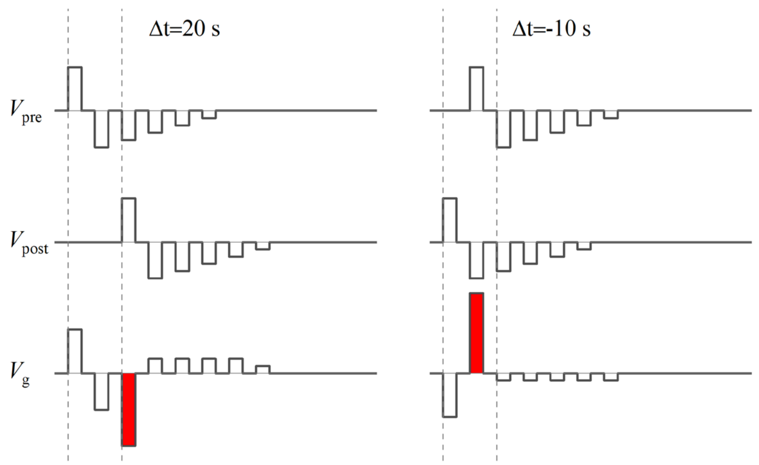

Fig. S18. Algorithm for the demonstration of STDP. Examples of input voltages for various relative spike timings of two neurons. The conductance change is measured at OCP condition.

SI References

1. T. Hallam, N. C. Berner, C. Yim, G. S. Duesberg, Strain, Bubbles, Dirt, and Folds: A Study of Graphene Polymer-Assisted Transfer. *Advanced Materials Interfaces* **1**, 1400115 (2014).

2. H. Vanselous, P. B. Petersen, Extending the Capabilities of Heterodyne-Detected Sum-Frequency Generation Spectroscopy: Probing Any Interface in Any Polarization Combination. *J. Phys. Chem. C* **120**, 8175–8184 (2016).

3. A. Adhikari, Accurate determination of complex $\chi^{(2)}$ spectrum of the air/water interface. *The Journal of Chemical Physics* **143**, 124707 (2015).

4. X. Liang, *et al.*, Toward Clean and Crackless Transfer of Graphene. *ACS Nano* **5**, 9144–9153 (2011).

5. J. W. Suk, *et al.*, Transfer of CVD-Grown Monolayer Graphene onto Arbitrary Substrates. *ACS Nano* **5**, 6916–6924 (2011).

6. M. S. Dresselhaus, A. Jorio, A. G. Souza Filho, R. Saito, Defect characterization in graphene and carbon nanotubes using Raman spectroscopy. *Phil. Trans. R. Soc. A.* **368**, 5355–5377 (2010).

7. S. Huh, *et al.*, UV/Ozone-Oxidized Large-Scale Graphene Platform with Large Chemical Enhancement in Surface-Enhanced Raman Scattering. *ACS Nano* **5**, 9799–9806 (2011).

8. L. G. Cançado, *et al.*, Quantifying Defects in Graphene via Raman Spectroscopy at Different Excitation Energies. *Nano Lett.* **11**, 3190–3196 (2011).

9. J.-H. Chen, *et al.*, Charged-impurity scattering in graphene. *Nature physics* **4**, 377–381 (2008).

10. Y. Wang, *et al.*, Direct Probe of Electrochemical Pseudocapacitive pH Jump at a Graphene Electrode**. *Angewandte Chemie International Edition* **62**, e202216604 (2023).

11. G. Froehlicher, S. Berciaud, Raman spectroscopy of electrochemically gated graphene transistors: Geometrical capacitance, electron-phonon, electron-electron, and electron-defect scattering. *Phys. Rev. B* **91**, 205413 (2015).

12. S. Das Sarma, S. Adam, E. H. Hwang, E. Rossi, Electronic transport in two-dimensional graphene. *Rev. Mod. Phys.* **83**, 407–470 (2011).

13. Y. Zhang, Y.-W. Tan, H. L. Stormer, P. Kim, Experimental observation of the quantum Hall effect and Berry's phase in graphene. *Nature* **438**, 201–204 (2005).

14. J. E. Lee, G. Ahn, J. Shim, Y. S. Lee, S. Ryu, Optical separation of mechanical strain from charge doping in graphene. *Nature Communications* **3**, 1024 (2012).

15. A. K. M. Newaz, Y. S. Puzyrev, B. Wang, S. T. Pantelides, K. I. Bolotin, Probing charge scattering mechanisms in suspended graphene by varying its dielectric environment. *Nat Commun* **3**, 734 (2012).

16. X. Jia, *et al.*, Kinetic Ionic Permeation and Interfacial Doping of Supported Graphene. *Nano Lett.* **19**, 9029–9036 (2019).

17. J. D. Miller, J. B. Hiskey, Electrokinetic behavior of fluorite as influenced by surface carbonation. *Journal of Colloid and Interface Science* **41**, 567–573 (1972).

18. S. Assemi, J. Nalaskowski, J. D. Miller, W. P. Johnson, Isoelectric Point of Fluorite by Direct Force Measurements Using Atomic Force Microscopy. *Langmuir* **22**, 1403–1405 (2006).

19. J. Cai, *et al.*, Wien effect in interfacial water dissociation through proton-permeable graphene electrodes. *Nat Commun* **13**, 5776 (2022).

20. A. Montenegro, *et al.*, Asymmetric response of interfacial water to applied electric fields. *Nature* **594**, 62–65 (2021).

21. T. Seki, *et al.*, Real-time study of on-water chemistry: Surfactant monolayer-assisted growth of a crystalline quasi-2D polymer. *Chem* **7**, 2758–2770 (2021).

22. Y.-C. Wen, *et al.*, Unveiling Microscopic Structures of Charged Water Interfaces by Surface-Specific Vibrational Spectroscopy. *Phys. Rev. Lett.* **116**, 016101 (2016).

23. Z. L. Mišković, P. Sharma, F. O. Goodman, Ionic screening of charged impurities in electrolytically gated graphene. *Phys. Rev. B* **86**, 115437 (2012).

24. F. Chen, J. Xia, N. Tao, Ionic Screening of Charged-Impurity Scattering in Graphene. *Nano Lett.* **9**, 1621–1625 (2009).

25. J. M. Gibbs-Davis, J. J. Kruk, C. T. Konek, K. A. Scheidt, F. M. Geiger, Jammed Acid−Base Reactions at Interfaces. *J. Am. Chem. Soc.* **130**, 15444–15447 (2008).

26. A. Citri, R. C. Malenka, Synaptic Plasticity: Multiple Forms, Functions, and Mechanisms. *Neuropsychopharmacol* **33**, 18–41 (2008).

27. H. Ye, *et al.*, High performance flexible memristors based on a lead free AgBiI4 perovskite with an ultralow operating voltage. *J. Mater. Chem. C* **8**, 14155–14163 (2020).

28. T. Fu, S. Fu, J. Yao, Recent progress in bio-voltage memristors working with ultralow voltage of biological amplitude. *Nanoscale* **15**, 4669–4681 (2023).

29. T. Sarkar, *et al.*, An organic artificial spiking neuron for in situ neuromorphic sensing and biointerfacing. *Nat Electron* **5**, 774–783 (2022).

30. G. Zhou, *et al.*, Volatile and Nonvolatile Memristive Devices for Neuromorphic Computing. *Advanced Electronic Materials* **8**, 2101127 (2022).

31. C.-Y. Wang, *et al.*, 2D Layered Materials for Memristive and Neuromorphic Applications. *Advanced Electronic Materials* **6**, 1901107 (2020).

32. P. Robin, *et al.*, Long-term memory and synapse-like dynamics in two-dimensional nanofluidic channels. *Science* **379**, 161–167 (2023).

33. G. Bi, M. Poo, Synaptic Modifications in Cultured Hippocampal Neurons: Dependence on Spike Timing, Synaptic Strength, and Postsynaptic Cell Type. *J. Neurosci.* **18**, 10464–10472 (1998).

34. H. Tian, *et al.*, Graphene Dynamic Synapse with Modulatable Plasticity. *Nano Lett.* **15**, 8013–8019 (2015).
